# The effectiveness of reduction in alcohol consumption achieved by the provision of non-alcoholic beverages associates with Alcohol Use Disorders Identification Test scores: a secondary analysis of a randomized controlled trial

**DOI:** 10.1186/s12916-024-03641-3

**Published:** 2024-09-30

**Authors:** Shohei Dobashi, Kyoko Kawaida, Go Saito, Yukiko Owaki, Hisashi Yoshimoto

**Affiliations:** 1 Institute of Health and Sports Science, University of Tsukuba, Tsukuba, Ibaraki Japan; 2 Research and Development Center for Lifestyle Innovation, University of Tsukuba, Tsukuba, Ibaraki Japan; 3 Department of General Medicine and Primary Care, Institute of Medicine, University of Tsukuba, Tsukuba, Ibaraki Japan

**Keywords:** Non-alcoholic beverages, Reduced alcohol consumption, Alcohol drinking behavior, Alcohol problem severity

## Abstract

**Background:**

The Alcohol Use Disorders Identification Test (AUDIT) is commonly used in clinical settings to assess the severity of alcohol-related problems, with the effectiveness of alcohol reduction interventions varying across this spectrum. In a recent study, we demonstrated that a 12-week intervention involving the provision of free non-alcoholic beverages reduced alcohol consumption among heavy drinkers for up to 8 weeks post-intervention. However, it remains unclear whether this effect was consistent across different AUDIT score ranges. Therefore, this secondary analysis aimed to examine whether the severity of alcohol-related problems, as indicated by AUDIT scores, influences the effectiveness of non-alcoholic beverage provision in reducing alcohol consumption.

**Methods:**

This was a single-center, open-label, randomized, parallel-group study. Participants were Japanese individuals who frequently consumed large quantities of alcohol (at least 40 g/day for men and 20 g/day for women) but were not diagnosed with alcohol dependence. Participants were randomly assigned to either an intervention or control group. The intervention group received free non-alcoholic beverages once every 4 weeks over a 12-week period (24 bottles of 350 mL per case, up to three cases per session, for a total of three sessions). Alcohol and non-alcoholic beverage consumption over the previous 4 weeks was tracked using a drinking diary. For this secondary analysis, participants were categorized into four groups based on their AUDIT scores (group 1: ≤ 7 points, group 2: 8–11 points, group 3: 12–14 points, and group 4: ≥ 15 points), and changes in alcohol consumption were compared across these groups in both the intervention and control participants.

**Results:**

The provision of non-alcoholic beverages significantly increased non-alcoholic beverage consumption in all groups. However, alcohol consumption was significantly reduced in the intervention groups compared to controls only in groups 1–3. The reduction in alcohol consumption was less pronounced in groups 3 and 4 compared to group 1 (both, *p* < 0.05). Importantly, the provision of non-alcoholic beverages did not lead to an increase in alcohol consumption, even among individuals with higher AUDIT scores.

**Conclusions:**

These findings suggest that individuals with higher AUDIT scores may experience a reduced benefit from a 12-week non-alcoholic beverage intervention in terms of alcohol consumption reduction. Nevertheless, this intervention appears to be a safe and effective strategy for reducing alcohol consumption in heavy drinkers who do not have alcohol dependence.

**Trial registration:**

UMIN UMIN000047949. Registered 4 June 2022.

**Supplementary Information:**

The online version contains supplementary material available at 10.1186/s12916-024-03641-3.

## Background

The AUDIT (Alcohol Use Disorders Identification Test) is a screening tool designed to assess patterns of alcohol consumption and identify potential alcohol-related problems [1]. Individuals with a high AUDIT score may be at increased risk of alcohol problem severity or alcohol consumption-related negative consequences such as physical health problems, impaired social or occupational functioning, and accidents or injuries [1]. Thus, it is necessary to investigate effective strategies to either prevent elevated AUDIT scores or improve alcohol use behavior in individuals who already have a high AUDIT score. A recent umbrella review demonstrated that brief interventions, cognitive behavioral therapy, and motivational interviews had a beneficial effect [2]. However, the brief interventions and motivational interviewing are considered more suitable for individuals with mild AUDIT severity, whereas cognitive behavioral therapy is suitable for individuals to those with higher severity [2]. These findings indicate that there is no universal strategy of reducing alcohol consumption and that selecting appropriate countermeasures according to the AUDIT severity might be important for improving alcohol-related problems.

We recently conducted a randomized controlled trial (RCT) showing that a 12-week intervention involving the provision of free non-alcoholic beverages significantly reduced alcohol consumption in heavy drinkers [3]. We also confirmed a significant replacement effect of non-alcoholic beverages by analyzing the correlation between changes in alcohol consumption and non-alcoholic beverage consumption [3]. Thus, the provision of non-alcoholic beverages is a new, potentially beneficial strategy for reducing alcohol consumption. However, a recent secondary analysis of our RCT revealed that non-alcoholic beverages significantly reduced alcohol consumption irrespective of gender, but the process by which this was achieved differed between men and women [5]. This result suggests that the influence of non-alcoholic beverage provision varies according to individual characteristics. 

A previous study showed that drinking non-alcoholic beverages could increase alcohol consumption by enhancing craving in individuals with harmful drinking habits [4]. Therefore, using non-alcoholic beverages to reduce alcohol consumption might not be a universal strategy, as with other interventions and be less, particularly in those with higher AUDIT scores. However, it remains unclear whether the benefits of non-alcoholic beverage provision disappeared depending on the alcohol problem severity.

We therefore tested whether AUDIT scores affect the degree to which providing non-alcoholic beverages influences alcohol consumption. We hypothesized that the magnitude of the reduction in alcohol consumption achieved by non-alcoholic beverage use would correlate with individuals’ pre-intervention AUDIT score.

## Methods

### Study design, procedure, and measurements

The current study presents expanded data from our recent study on how the free provision of non-alcoholic beverages impacted alcohol consumption [3, 5]. That study was conducted after approval by the ethics committee of the University of Tsukuba (Notification Number G299) and was registered as UMIN000047949 (Randomized controlled study on the impact of serving non-alcoholic beverages on alcohol consumption). We adopted a single-center, open-label, randomized, parallel-group design. Individuals who are at least 20 years old and drank on 4 or more days per week, with alcohol consumption of at least 40 g for men or 20 g for women on each of those days, were enrolled. This volume of alcohol consumption was defined as medium risk of alcohol-related health problems according to the definition of the World Health Organization (WHO) [6]. Although this is the first RCT study whether non-alcoholic beverage provisions on alcohol consumption, we could not ignore the possibility that non-alcoholic beverage provision exacerbated heavy drinking in the drinkers. Depending on the case, the intervention would exacerbate the symptoms of liver diseases which often occurred by heavy drinking. Therefore, it was necessary to exclude in advance those at risk so that even if adverse effects from the provision of non-alcoholic beverages were to occur, they would not cause irreversible adverse effects on the body. Considering these concerns and smooth implementation of research, the exclusion criteria were consumption of non-alcoholic beverages at least twice per month, past history of liver disease, current pregnancy or nursing, alcohol dependence, lack of consent for the use of LINE® (a messaging application adopted widely throughout Japan that can be used on personal computers or smartphones; LINE Corp., Shinjuku-ku, Tokyo, Japan), and inability to understand the study explanation or answer the pre-intervention online survey, both of which were written only in Japanese. Prior to the randomization, a 2-h face-to-face orientation session was held that included a well-experienced physician interview regarding alcohol dependence, based on the International Statistical Classification of Diseases and Related Health Problems-10 (ICD-10). We then excluded individuals with alcohol dependence because it has been suggested that their use of non-alcoholic beverages may enhance alcohol craving and stimulate their desire to drink, which may increase the risk of drinking relapse [4].

Written informed consent was obtained from those who passed the screening during the orientation session. During this briefing, participants were asked to complete a questionnaire regarding factors such as age, sex, race, marital status, highest level of education, employment status, household income, smoking history, and subjective view of health, as well as the Alcohol Quality of Life Scale (AQoLS) [7] and questions related to drinking, specifically the number of binge-drinking episodes within the past month and the items in the AUDIT [1]. In addition, height and body weight were measured and a saliva test was administered to assess the activity of genes related to alcohol metabolism, such as alcohol dehydrogenase 1B (*ADH1B*) and aldehyde dehydrogenase 2 (*ALDH2*) [8].

Following the briefing, simple randomization using a random number table was used to randomly allocate the participants to the non-alcoholic beverage provision (intervention) group or the control group [9]. Free non-alcoholic beverages were provided once every 4 weeks (three times in total). Each case included 24 350-mL bottles. Up to three cases were provided at a time. Thus, the participants in the intervention group received a maximum of 216 350 mL non-alcoholic beverages. In this study, we used non-alcoholic beverages that contained 0.00% alcohol by volume, had a taste similar to that of alcoholic beverages, and were designed or suggested for individuals aged 20 years or older as defined by the Japanese Alcoholic Beverage Advertising Review Committee. The beverages were selected separately by each participant depending on their preferences. Since the purpose of this study was to investigate whether increased availability of non-alcoholic beverages would change the amount of alcohol consumption, there were no stipulations regarding how to drink (amount or frequency). No other (psychological and/or educational) interventions were not conducted for both groups in this study. Participants in both groups were asked to record their consumption of alcoholic and non-alcoholic beverages in a calendar-format drinking diary every day, from 2 weeks before the start of the 12-week intervention to 8 weeks after its completion. All participants were also required to submit the drinking diary to the research staff every 4 weeks using LINE®. After the briefing, the study participants were contacted only by phone or via internet. At the end of the study, a gift card worth 10,000 yen (approximately 73.67 US dollars) was given to all participants as a reward. In addition, each participant in the control group received up to five cases of non-alcoholic beverages of their choice, but not received any intervention throughout the study period.

### Data analysis

**Fig. 1:**
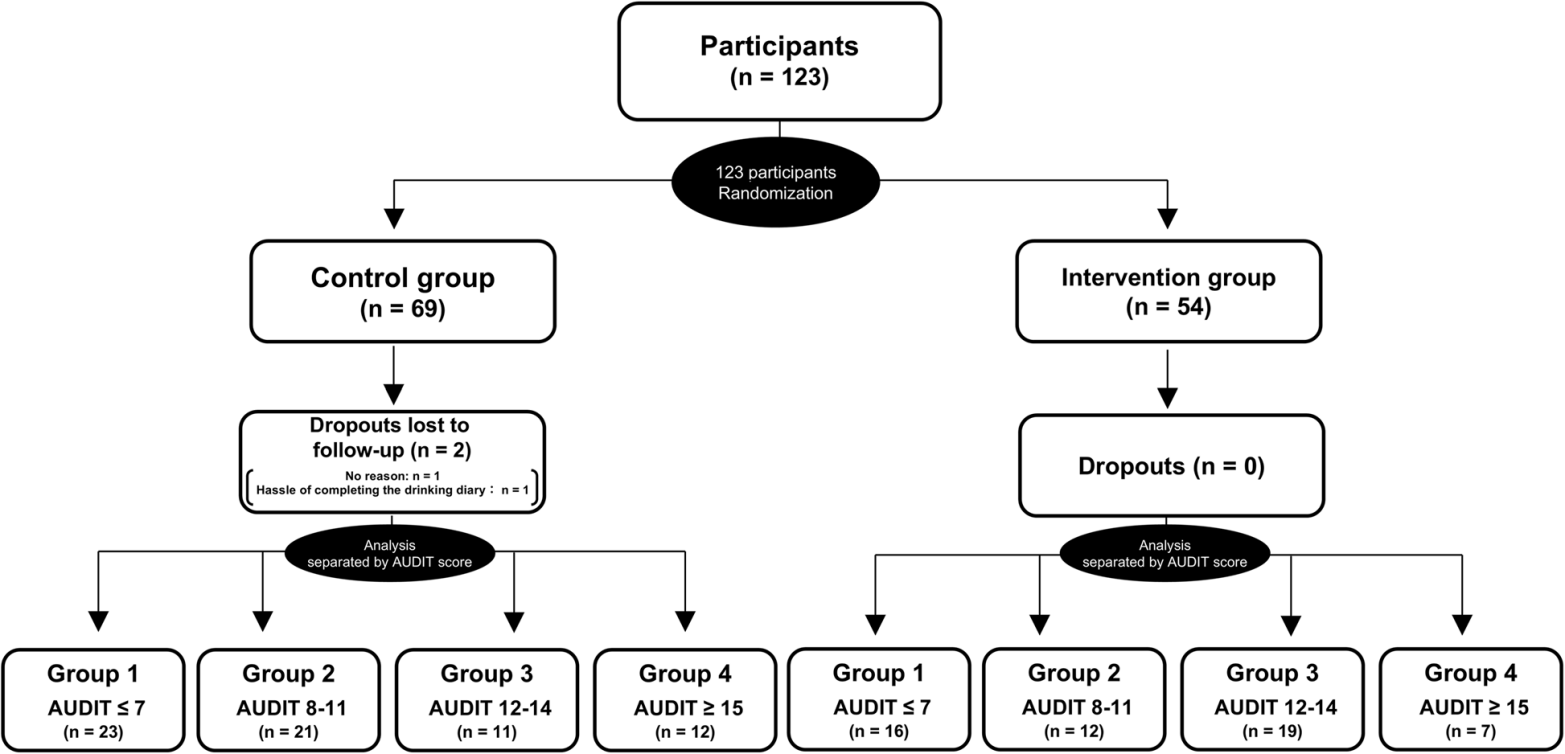
Study flow diagram

Although the amounts of alcoholic and non-alcoholic beverages consumed were obtained every 4 weeks, for practical purposes the mean amounts of alcoholic and non-alcoholic beverages consumed per 4 weeks were used in the analyses. We calculated the amount of alcohol consumption from the drinking diary data using the following formula: “consumption (mL) × alcohol concentration (%, v/v) × specific gravity (0.8)/100” [10]. We also calculated drinking frequency (drinking days per 4 weeks) and alcohol consumption on the day of drinking [5]. AUDIT is used as a screen for the full spectrum of risky drinking [11]. The WHO defines AUDIT scores of 7 or lower as a low risk of alcohol-related health problems, 8–15 as medium risk, and 16 or higher as high risk, but the relationship between AUDIT scores and alcohol-related problems is not universal and varies by ethnicity and race [1]. Previous studies in Japan have suggested that the AUDIT cutoff points for dangerous drinking is 8, harmful drinking is 12, and potential alcohol use disorder is 15 [12–15]. Thus, we divided the participants into four groups for secondary analysis according to their AUDIT scores. Briefly, we defined an AUDIT score of 7 or lower as group 1, 8–11 as group 2, 12–14 as group 3, and 15 or higher as group 4 (Fig. 1). The participants in group 4 may have had alcohol use disorder in terms of AUDIT score, but they were not diagnosed with alcohol dependence in physician interviews based on the ICD-10 prior to the start of this study. Therefore, this secondary analysis means that was an examination of the differences in classification based on AUDIT scores on the effect of non-alcoholic beverage provision on reducing alcohol consumption in heavy drinkers without alcohol dependence. In both the control and intervention groups, we expressed changes in alcohol consumption between the intervention and follow-up periods as percent changes from baseline levels [5]. Percent changes in non-alcoholic beverage consumption could not be calculated if participants did not consume non-alcoholic beverages at baseline. Hence, the consumption of non-alcoholic beverages was calculated based on the amount (mL) of non-alcoholic beverages consumed.

Furthermore, to examine whether alcohol consumption was reduced by replacing alcoholic beverages with non-alcoholic ones, the correlation between changes in alcohol consumption and the consumption of non-alcoholic beverages was analyzed.

### Statistical analysis

The available data of all participants were included according to the original allocation in an intention-to-treat analysis. The unsubmitted data of the participants who dropped out were treated as missing data. The normality of the data was evaluated by the Kolmogorov–Smirnov test. Intergroup comparisons of baseline data in each AUDIT group were performed by the *t*-test in cases of normal distribution and by the Mann–Whitney *U* test in cases of non-normal distribution. The chi-square test or Fisher’s exact probability test was used to compare categorical variables. Two-way repeated measures analysis of variance (ANOVA) was used to analyze longitudinal changes in the consumption of alcoholic and non-alcoholic beverages within each AUDIT group in both control and intervention participants. The normality assumption was not met for all variables. However, the homogeneity of the variance of the data was confirmed for both variables using Levene’s test, and therefore the ANOVA results were accepted with reference to a preceding study [16]. The post hoc Bonferroni’s test was performed to analyze time course changes within a single group, as well as intergroup differences at individual time points. The Kruskal–Wallis test was used to analyze changes from baseline levels in the consumption of alcoholic and non-alcoholic beverages between intervention groups in the four AUDIT groups during the study, including the intervention periods. Correlations were evaluated using Spearman’s rank correlation coefficient. The significance level was 5%. The effect size was described as partial *η* squared (*η*_p_^2^) and 0.01, 0.06, and 0.14 were considered as small, medium, and large effect sizes, respectively [17]. GraphPad Prism v. 9.0 (GraphPad Inc., La Jolla, CA, USA) and Stata 18 SE for Windows (Stata Corp., College Station, TX, USA) were used for all analyses.

## Results

One-hundred twenty-three people participated this study, and all of them were randomized. After the randomization, we confirmed that there were no significant differences in their basic characteristics, as previously shown in our original study [3]. Moreover, the baseline characteristics of participants even after further divided into the four AUDIT groups are also not different (Table 1 and Supplementary Material 1: Table S1).

**Table 1:** Baseline characteristics of participants in this secondary analysis

	Group 1 (*n* = 39)		*P*-value	Group 2 (*n* = 33)		*P*-value	Group 3 (*n* = 30)		*P*-value	Group 4 (*n* = 19)		*P*-value
	Control (*n* =23, 59.0%)	Intervention (*n* = 16, 41.0%)		Control (*n* =21, 63.6 %)	Intervention (*n* = 12, 36.4%)		Control (*n* = 11, 36.7%)	Intervention (*n* = 19, 63.3%)		Control (*n* =12, 63.1%)	Intervention (*n* = 7, 36.9%)	
Age (years, SD)	48.0 (10.6)	46.5 (10.0)	0.67^a^	49.9 (9.5)	43.6 (11.3)	0.10^a^	44.4 (10.7)	53.4 (9.6)	0.03^a^	45.1 (7.3)	42.9 (9.1)	0.57^a^
Female (number of participants, %)	17 (73.9)	11 (68.8)	0.73^b^	15 (71.4)	8 (66.7)	0.78^b^	5 (45.5)	13 (31.6)	0.45^d^	3 (25.0)	3 (42.9)	0.42^d^
Height (cm, SD)	163.3 (7.8)	163.3 (6.2)	0.99^a^	163.9 (8.6)	166.0 (10.0)	0.53^a^	169.2 (10.5)	167.0 (9.0)	0.55^a^	169.8 (9.0)	165.9 (7.8)	0.36^a^
Body weight (kg, SD)	59.7 (13.5)	62.1 (16.8)	0.63^a^	61.7 (14.7)	63.7 (13.6)	0.71^a^	66.9 (11.0)	66.9 (10.5)	0.99^a^	74.4 (13.2)	63.1 (14.5)	0.10^a^
AUDIT (points, median, IQR)	6.0 (2.0)	6.0 (1.0)	0.84^c^	8.0 (1.0)	9.0 (2.3)	0.16^c^	13.0 (2.0)	13.0 (2.0)	0.92^c^	17.0 (3.3)	18.0 (2.0)	0.64^c^
Binge drinking (times/month, median, IQR)	1.0 (3.5)	2.0 (9.3)	0.24^c^	2.0 (13.0)	1.0 (2.3)	0.06^c^	15.0 (18.5)	5.0 (21.0)	0.63^c^	10.0 (12.5)	20 (12.0)	0.23^c^
Number of HED (times/4 weeks, median, IQR)	0.0 (1.0)	1.0 (1.0)	0.66^c^	1.0 (3.0)	1.0 (3.3)	0.85^c^	7.0 (16.5)	10.0 (11.0)	0.89^c^	7.0 (18.3)	15.0 (14.5)	0.37^c^

*AUDIT* Alcohol Use Disorders Identification Test, *HED* Heavy episodic drinking, *SD* Standard deviation, *IQR* Interquartile range

^a^t-test

^b^Chi-square test

^c^Mann–Whitney U test

^d^Fisher's exact probability test

**Fig. 2:**
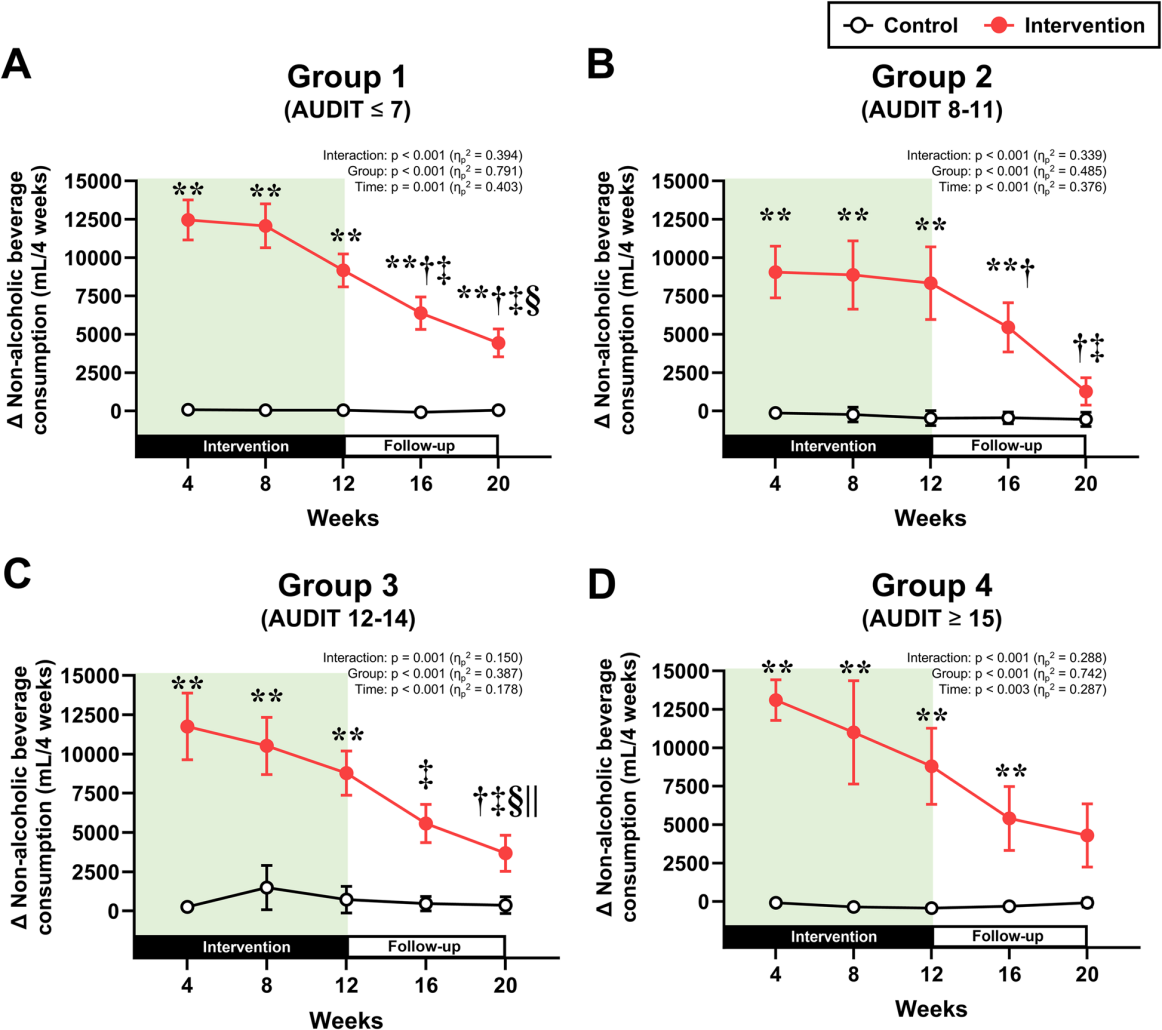
Non-alcoholic beverage consumption. Changes in non-alcoholic beverage consumption from baseline (week 0) in the four groups based on AUDIT scores: **A** group 1, **B** group 2, **C** group 3, and **D** group 4. **p < 0.01 vs. the control group at the same time point. ^†^*p* < 0.05 vs. week 4, ^‡^*p* < 0.05 vs. week 8, ^§^*p* < 0.05 vs. week 12, ^||^*p* < 0.05 vs. week 16

**Fig. 3:**
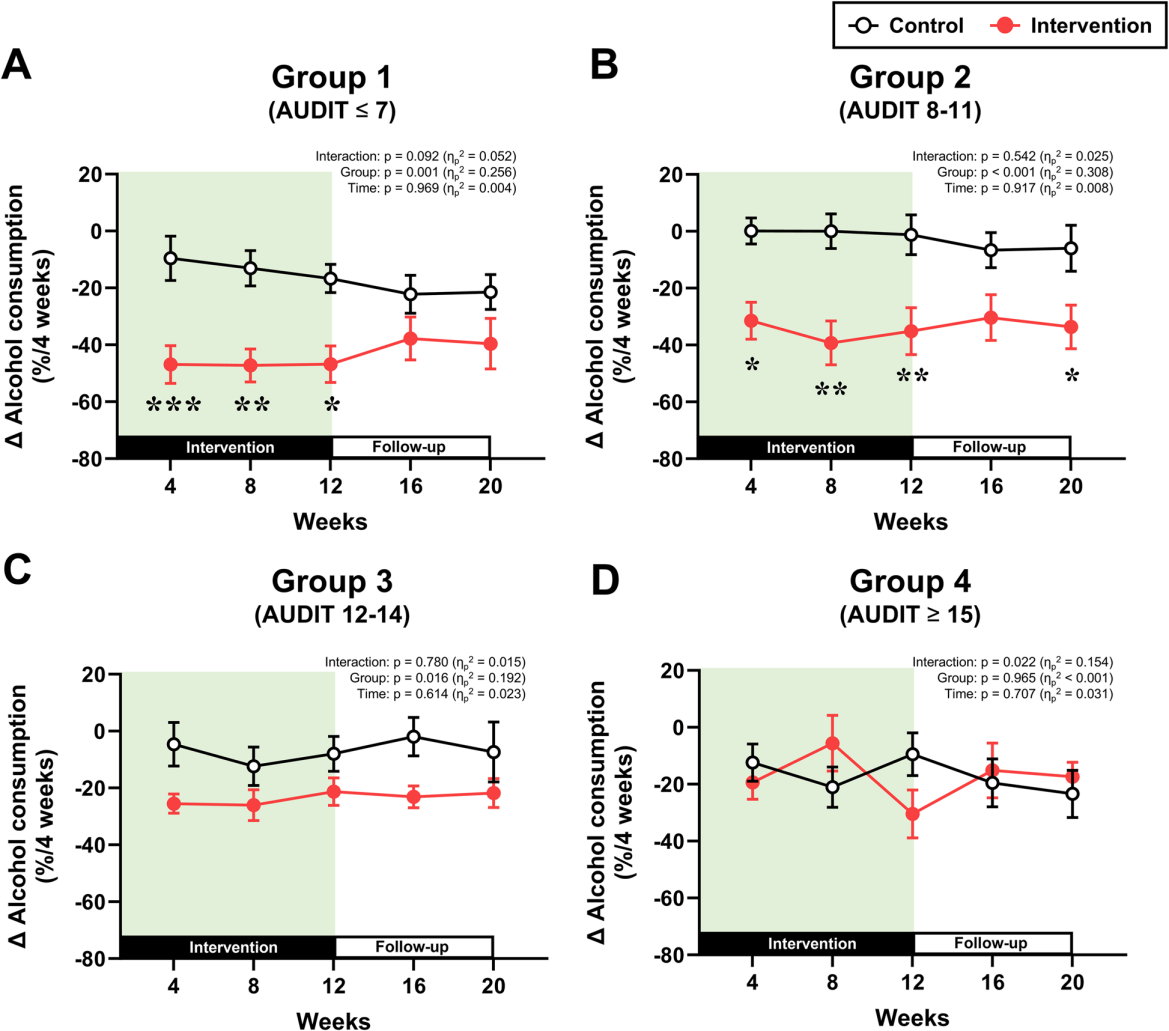
Alcohol consumption. Changes in alcohol consumption from baseline (week 0) in the four groups based on AUDIT scores: **A** group 1, **B** group 2, **C** group 3, and **D** group 4. ^***^p < 0.001, ^**^p < 0.01, ^*^p < 0.05 vs. the control group at the same time point

The longitudinal changes in non-alcoholic beverage consumption and alcohol consumption, which were separately analyzed by AUDIT group, are presented in Figs. 2 and 3, respectively (detailed numeric data about the figures are also represented in Supplementary Material 1: Table S2). Two-way repeated measures ANOVA revealed interactions of group and time were observed for the changes in non-alcoholic beverage consumption in all AUDIT groups (all *p* < 0.05, Fig. 2A–D). In the intervention group, non-alcoholic beverage consumption increased after the start of the intervention in all AUDIT groups (Fig. 2A–D). Although this consumption gradually decreased after week 4 in all AUDIT groups, it remained significantly greater than that in the control group in all AUDIT groups throughout the 12-week intervention (*p* < 0.05). The significant increase in non-alcoholic beverage consumption lasted as long as week 20 in groups 1–3, but not in group 4. For the percent changes in alcohol consumption from week 0, main effect of group was observed in groups 1–3, but not in group 4 (Fig. 3A–D). Post hoc analysis demonstrated that the changes in alcohol consumption at weeks 4, 8, and 12 were significantly lower in the intervention group than in the control group only in groups 1–3 (all *p* < 0.05). Nevertheless, no significant differences between the control and intervention groups during follow-up period in all AUDIT groups.

**Fig. 4:**
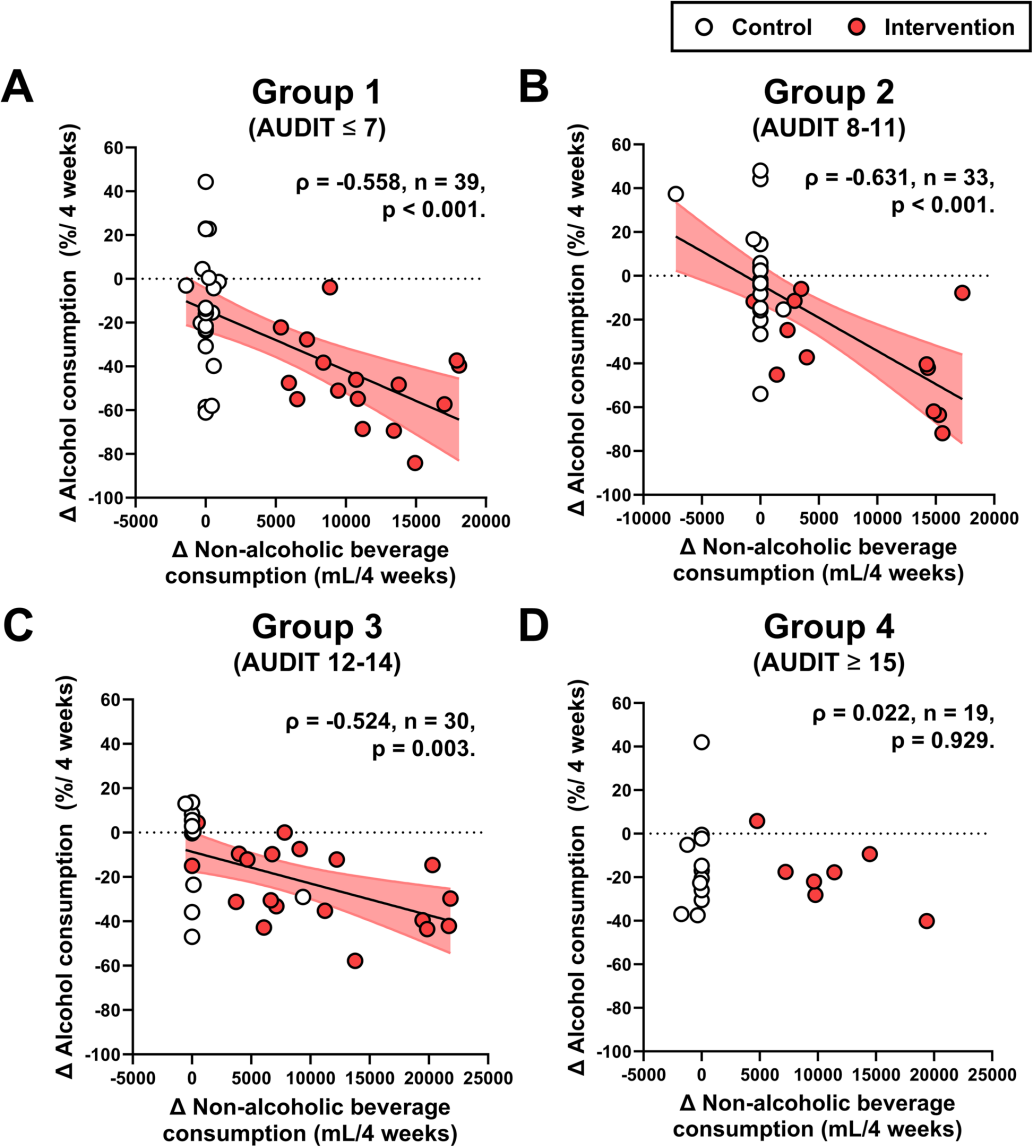
Correlation coefficients between changes in alcohol consumption and non-alcoholic beverage consumption during the intervention period. **A** Group 1, **B** group 2, **C** group 3, and **D** group 4

Figure 4 shows the correlation coefficients between the percent mean changes in alcohol consumption and the mean changes in non-alcoholic beverage consumption during the 12-week intervention. Although significant negative correlation coefficients between alcohol consumption and non-alcoholic beverage consumption were observed in groups 1–3 (*ρ* =  − 0.558, − 0.631, − 0.524, respectively. All *p* < 0.01, Fig. 4A–C), no significant relationship was seen in group 4 (Fig. 4D).

**Fig. 5:**
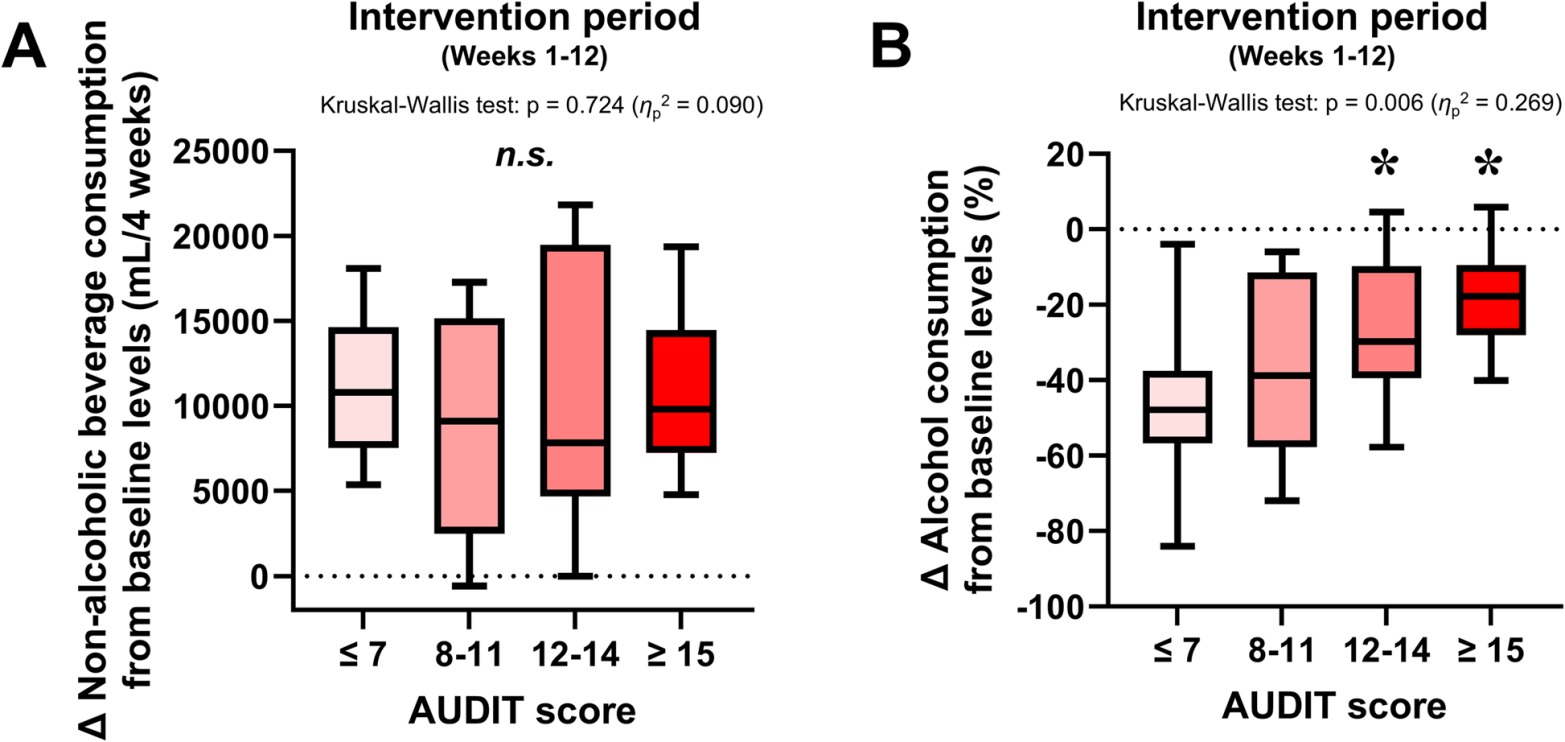
Changes from baseline in non-alcoholic beverage consumption and alcohol consumption, compared by AUDIT group. Mean changes in non-alcoholic beverage (**A**) and alcohol (**B**) consumption during the intervention period (weeks 1–12). Each box represents the interquartile range (IQR), with the lower edge of the box indicating the 25th percentile and the upper edge indicating the 75th percentile. The whiskers extend from the box to the minimum and maximum values. n.s., not significant. *p < 0.05 vs. group 1 (AUDIT score of 7 or lower)

The mean changes in alcohol consumption and non-alcoholic beverage consumption relative to baseline values in the intervention group during the 12-week intervention period are shown in Fig. 5, which was not included the data in the control group. There were no significant differences between the AUDIT groups regarding changes in non-alcoholic beverage consumption (*p* = 0.724, Fig. 5A). In contrast, Kruskal–Wallis test for the magnitude of the reduction in alcohol consumption during the intervention period revealed significant (*p* = 0.006) and post hoc analysis demonstrated that the changes in alcohol consumption was significantly attenuated in groups 3 and 4 compared with group 1 (both *p* < 0.05, Fig. 5B).

**Fig. 6:**
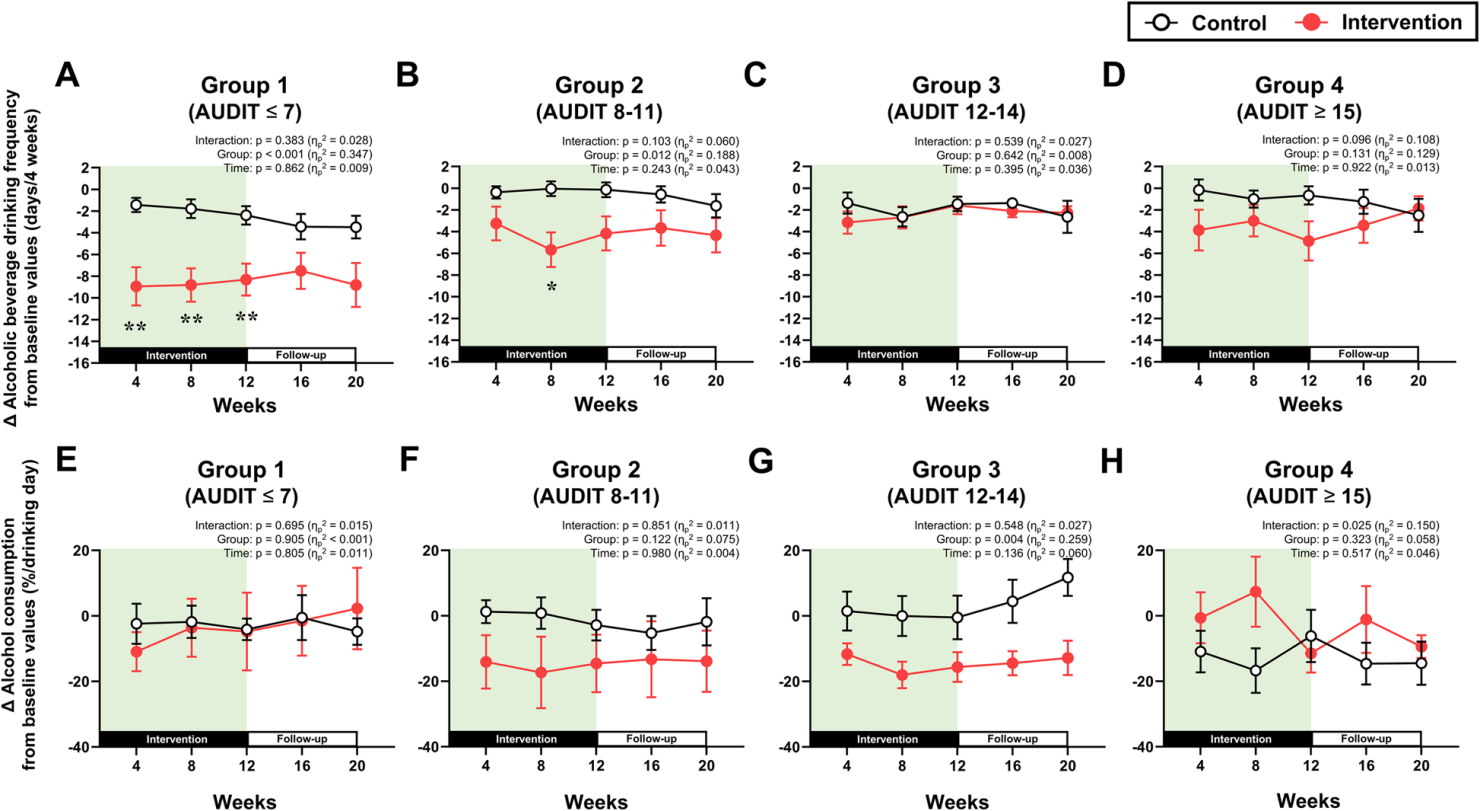
Alcoholic beverage drinking frequency and alcohol consumption on drinking days. **A**–**D** Changes in alcoholic beverage drinking frequency from baseline (week 0) in groups 1–4. **E**–**H** Percent changes in alcohol consumption from baseline (week 0) in group 1–4. **p < 0.01, *p < 0.05 vs. the control group at the same time point

The longitudinal changes in alcoholic beverage drinking frequency and alcohol consumption on the day of drinking, both of which were separately analyzed in the four AUDIT groups, are presented in Fig. 6. The alcoholic beverage drinking frequency was significantly decreased in groups 1 and 2, but not in group 3 or 4 (Fig. 6A–D). The relative change in alcohol consumption on drinking days was decreased by the intervention only in group 3 (Fig. 6E–H).

## Discussion

In this study, we examined whether the provision of non-alcoholic beverages affected alcohol consumption differently based on the AUDIT score before the start of the intervention. We found that providing non-alcoholic beverages significantly reduced alcohol consumption only in individuals with AUDIT scores of 14 or lower, and the magnitude of this reduction decreased as the AUDIT score increased. Furthermore, while the reduced alcohol consumption was associated with a decrease in drinking frequency in individuals with low AUDIT scores (groups 1 and 2), it was linked to a decrease in the amount of alcohol consumed per drinking day in participants with high AUDIT scores (group 3). These findings suggest that the AUDIT score influences both the impact of non-alcoholic beverage provision on alcohol consumption and with changes in drinking behavior.

Non-alcoholic beverage consumptions significantly increased and remained elevated in the intervention group compared with the control group throughout the study period irrespective of AUDIT score. While the reason for long-lasting higher values of non-alcoholic beverage consumption even in follow-up period remain unknown, it is possible that some of the non-alcoholic beverages provided at weeks 0, 4, and 8 were consumed by the participants after week 12 [3]. This secondary analysis was the first to compare the non-alcoholic beverage consumption across different AUDIT classifications, and the results suggest that alcohol problem severity does not affect non-alcoholic beverage consumption.

We recently demonstrated that the provision of non-alcoholic beverages significantly reduced alcohol consumption in heavy drinkers, irrespective of gender [5]. In contrast, although we observed significantly reduced alcohol consumption in AUDIT groups 1–3, we found no reduction in group 4. To further explore this, we analyzed the correlation between changes in alcohol consumption and non-alcoholic beverage consumption to assess the replacement effect of non-alcoholic beverages on alcoholic beverages during the 12-week intervention [3]. Significant correlation coefficients were observed in groups 1–3, but not in group 4. Since we also observed no significant changes in non-alcoholic beverage consumption between groups 1–4, it raises the possibility that individuals with an AUDIT score of 15 points or higher are less likely to experience a replacement effect from the provision of alcoholic beverages. In other words, this population may indicate “replacement resistance”. A previous study also showed that when individuals with significant alcohol dependence consume non-alcoholic beverages, these do not replace the alcoholic beverages they drink [4], and this supports the new hypothesis derived from our results.

Interestingly, the reduction in alcohol consumption from the intervention was significantly attenuated in group 3–4 compared with group 1. Previous studies have not yielded consistent results in this regard: While one study found that some interventions for reducing alcohol consumption (i.e., screening and brief intervention) were more effective for the individuals with higher AUDIT scores than those lower AUDIT scores [18], another study did not [2]. This is the first study to examine the effect of non-alcoholic beverage provision according to AUDIT score, but the results do not suggest that such interventions are effective in decreasing alcohol consumption among people with high AUDIT scores.

However, it remains unclear why higher AUDIT scores attenuated the reduction in alcohol consumption associated with non-alcoholic beverage provision. It was previously shown that when non-alcoholic beverages were offered, reduced alcohol consumption was achieved in women primarily through decreased drinking frequency, but in men mainly through a decrease in the amount of alcohol consumed on a given drinking day [5]. Thus, the drinking frequency and alcohol consumption on each drinking day were both calculated in this study. The intervention significantly decreased the drinking frequency in groups 1 and 2, but not in group 3 or 4. On the other hand, only group 3 showed a significant decrease in alcohol consumption on each drinking day. Since the groups in this study had similar compositions in terms of men and women, these differences in the process by which the intervention reduced alcohol consumption may be due not to gender differences, but rather to the level of alcohol problem severity. Hence, it is possible that when the AUDIT score is low, a reduction in alcohol consumption can be achieved by decreasing drinking frequency, while as the AUDIT score becomes higher, it can be accomplished by reducing the amount of alcohol consumed on drinking days. Then, as the AUDIT score became even higher in this study, it was no longer possible to reduce the amount of alcohol consumed on drinking days. Altogether, we speculate that these shifts in drinking behavior in response to non-alcoholic beverage provision occurred in relation to increasing AUDIT scores and that this may have been responsible for the differences in the degree of alcohol reduction (Table 2). 

Nevertheless, the mechanisms underlying these differences in drinking behavior remain unclear. Previous studies have reported that patients with high AUDIT scores may also suffer from sleep disorders, that they drink to induce sleep [19, 20], and that craving for alcohol is increased due to dysregulation of appetite-regulating hormones [21, 22]. A previous study in monkeys has also indicated that long-term alcohol consumption decreases the secretion of dopamine, which is involved in the reward system [23]. These findings raise the possibility that the increase in non-alcoholic beverage consumption did not contribute to the reduction of alcohol intake in individuals with high AUDIT scores due to the involvement of these complex factors. Further studies are needed to elucidate this issue using behavioral and neurobiological approaches.

**Table 2:**
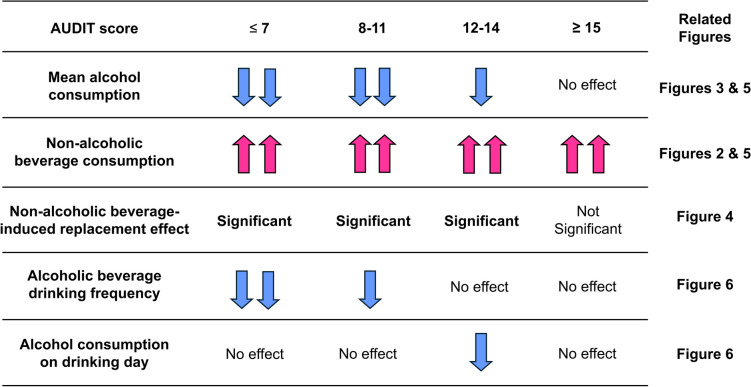
The summary of influence of AUDIT score on changes in drinking behavior induced by the provision of non-alcoholic beverages

The blue and red arrows indicate a decrease or increase, respectively, with two arrows indicating a greater difference. The table suggests that shift changes in drinking behaviors in response to non-alcoholic beverage provision occurred dependent on increasing AUDIT scores and that this may have been responsible for the differences in the degree of alcohol reduction

Based on the results of this study, the provision of non-alcoholic beverages is an effective intervention for reducing alcohol consumption among those with low AUDIT scores (14 points or lower). Irrespective of AUDIT score, however, no adverse effects, such as increased alcohol consumption (which could be caused by increased craving), were observed when non-alcoholic beverages were offered. A recent cross-sectional study showed similar results [24], suggesting that interventions involving non-alcoholic beverages may be an effective first step to reduce alcohol consumption. Individuals who do not benefit may then require alternative approaches, such as face-to-face brief counseling interventions, including motivational interviewing and cognitive behavioral therapy according to clinical guidelines, the use of electronic devices to provide information and advice, and the creation of social and physical environments that reduce the number of drinking occasions and the number of drinks consumed on each occasion [25–29].

While this secondary analysis demonstrated important findings, we must acknowledge some limitations. The main limitations of this RCT have already been reported in a previous study [3]. In particular, the study analyzed only individuals who were confirmed not to have alcohol dependence. Therefore, it is unclear whether these results can be applied to individuals with alcohol dependence or those at a high risk of alcohol-related health problems. Moreover, as we used only self-reported diaries to obtain the amount of alcoholic and non-alcoholic beverage consumption, without objective biomarkers of alcohol use, caution is required when interpreting the results. Furthermore, the number of participants for each group in this study was quite small because the intervention and control participants in the RCT [3] were further divided according to the AUDIT score. Considering that the significant reduction in alcohol consumption remained until 20 weeks in our original RCT [3], the disappeared significant reduction was observed during the follow-up period might be due to small sample size. Moreover, we cannot deny the possibility that the disappearance of the significant reduction in alcohol consumption associated with non-alcoholic beverage provision might be due to low statistical power stemming from the quite small sample size. Larger studies, especially involving individuals with higher AUDIT scores, should be performed in the future. Nevertheless, we believe that the present study constitutes a firm foundation for solving alcohol-related problems.

## Conclusions

Our findings demonstrate that providing non-alcoholic beverages for 12 weeks reduced alcohol consumption, but this effect was attenuated by high AUDIT scores. However, even among individuals with high AUDIT scores, the provision of non-alcoholic beverages did not promote increased alcohol consumption, suggesting that this intervention may be a relatively simple and effective strategy for reducing alcohol consumption.

## Supplementary Information

Supplementary Material 1. Table S1. Baseline characteristics not shown in Table 1. Table S2. Numeric data for the longitudinal changes from baseline values in non-alcoholic beverage and alcohol consumptions, drinking frequency, and alcohol consumption on drinking day.

## Data Availability

No datasets were generated or analysed during the current study.

## Abbreviations

ADH1B: Alcohol dehydrogenase 1B
ALDH2: Aldehyde dehydrogenase 2
ANOVA: Analysis of variance
AQoLS: Alcohol Quality of Life Scale
AUDIT: Alcohol Use Disorders Identification Test
HED: Heavy episodic drinking
WHO: World Health Organization
