# Supplementary material for: The effectiveness of reduction in alcohol consumption achieved by the provision of non-alcoholic beverages associates with Alcohol Use Disorders Identification Test scores: a secondary analysis of a randomized controlled trial
Source: BMC Med. 2024 Sep 30;22:424. doi: 10.1186/s12916-024-03641-3 (PMC11441128; doi:10.1186/s12916-024-03641-3)
Supplement: Supplementary file 1 — Supplementary Material 1. Table S1. Baseline characteristics not shown in Table 1. Table S2. Numeric data for the longitudinal changes from baseline values in non-alcoholic beverage and alcohol consumptions, drinking frequency, and alcohol consumption on drinking day. [file 12916_2024_3641_MOESM1_ESM.docx]

Table S1　Baseline characteristics not shown in Table 1.

|  | Group 1 (n = 39) | | P-value | Group 2 (n = 33) | | P-value | Group 3 (n = 30) | | P-value | Group 4 (n = 19) | | P-value |
| --- | --- | --- | --- | --- | --- | --- | --- | --- | --- | --- | --- | --- |
|  | Control (n = 23, 59.0%) | Intervention (n = 16, 41.0%) |  | Control (n = 21, 63.6 %) | Intervention (n = 12 , 36.4 %) |  | Control (n = 11, 36.7 %) | Intervention (n = 19 , 63.3%) |  | Control (n = 12, 63.1 %) | Intervention (n = 7 , 36.9%) |  |
| Japanese (number of participants, %) | 23 (100) | 16 (100) | N.D. | 21 (100) | 12 (100) | N.D. | 11 (100) | 18 (94.7) | 1.00^a^ | 12 (100) | 7 (100) | N.D. |
| Married (number of participants, %) | 17 (73.9) | 12 (52.2) | 0.62^a^ | 18 (85.7) | 8 (66.7) | 0.38^a^ | 7 (63.6) | 12 (63.2) | 1.00^a^ | 9 (75.0) | 5 (71.4) | 1.00^a^ |
| Highest level of education (number of participants, %) | |  | 0.48^b^ |  |  | 0.73^b^ |  |  | 0.75^b^ |  |  | 0.17^b^ |
| Junior high school | 0 (0.0) | 0 (0.0) |  | 0 (0.0) | 1 (8.3) |  | 0 (0.0) | 0 (0.0) |  | 0 (0.0) | 0 (0.0) |  |
| High school | 3 (13.0) | 6 (37.5) |  | 2 (9.5) | 1 (8.3) |  | 0 (0.0) | 3 (15.8) |  | 1 (8.3) | 3 (42.9) |  |
| Vocational school | 4 (17.4) | 1 (6.3) |  | 2 (9.5) | 2 (16.7) |  | 1 (9.1) | 1 (5.3) |  | 1 (8.3) | 0 (0.0) |  |
| Junior college, specialized vocational high school | 5 (21.7) | 2 (12.5) |  | 4 (19.1) | 1 (8.3) |  | 2 (18.2) | 2 (10.5) |  | 0 (0.0) | 1 (14.3) |  |
| College | 9 (39.1) | 6 (37.5) |  | 7 (33.3) | 5 (41.7) |  | 6 (54.5) | 9 (47.4) |  | 5 (41.7) | 2 (28.6) |  |
| Graduate school | 2 (8.7) | 1 (6.3) |  | 6 (28.6) | 2 (16.7) |  | 2 (18.2) | 4 (21.1) |  | 5 (41.7) | 1 (14.3) |  |
| Employed (number of participants, %) | 22 (95.7) | 15 (93.8) | 0.66^a^ | 21 (100) | 10 (83.4) | 0.13^a^ | 11 (100) | 19 (100) | N.D. | 12 (100) | 7 (100) | N.D. |
| Household income (number of participants, %) | |  | 0.41^b^ |  |  | 0.17^b^ |  |  | 0.68^b^ |  |  | 1.00^b^ |
| Less than 1 million yen | 0 (0.0) | 0 (0.0) |  | 0 (0.0) | 1 (8.3) |  | 0 (0.0) | 0 (0.0) |  | 0 (0.0) | 0 (0.0) |  |
| 1 to 2 million yen | 0 (0.0) | 0 (0.0) |  | 0 (0.0) | 0 (0.0) |  | 0 (0.0) | 0 (0.0) |  | 0 (0.0) | 0 (0.0) |  |
| 2 to 3 million yen | 1 (4.3) | 2 (12.5) |  | 0 (0.0) | 1 (8.3) |  | 0 (0.0) | 0 (0.0) |  | 0 (0.0) | 0 (0.0) |  |
| 3 to 4 million yen | 2 (8.7) | 2 (12.5) |  | 1 (4.8) | 0 (0.0) |  | 2 (18.2) | 2 (10.5) |  | 0 (0.0) | 0 (0.0) |  |
| 4 to 5 million yen | 2 (8.7) | 4 (25.0) |  | 4 (19.1) | 0 (0.0) |  | 1 (9.1) | 3 (15.8) |  | 1 (8.3) | 1 (14.3) |  |
| 5 to 8 million yen | 6 (26.1) | 1 (6.3) |  | 6 (28.6) | 1 (8.3) |  | 3 (27.3) | 5 (26.3) |  | 4 (33.3) | 2 (28.6) |  |
| 8 to 10 million yen | 7 (30.4) | 3 (18.7) |  | 3 (14.3) | 3 (25.0) |  | 3 (27.3) | 2 (10.5) |  | 3 (25.0) | 1 (14.3) |  |
| More than 10 million yen | 5 (21.7) | 4 (25.0) |  | 7 (33.3) | 6 (50.0) |  | 2 (18.2) | 7 (36.8) |  | 4 (33.3) | 3 (42.9) |  |
| Smoking history (number of participants, %) | |  | 0.44^b^ |  |  | 0.40^b^ |  |  | 0.64^b^ |  |  | 0.53^b^ |
| Smoker | 2 (8.7) | 3 (18.7) |  | 4 (19.1) | 0 (0.0) |  | 3 (27.3) | 8 (42.1) |  | 2 (16.7) | 0 (0.0) |  |
| Ex-smoker | 6 (26.1) | 2 (12.5) |  | 7 (33.3) | 5 (41.7) |  | 5 (45.4) | 5 (26.3) |  | 4 (33.3) | 4 (57.1) |  |
| Non-smoker | 15 (65.2) | 11 (68.8) |  | 10 (47.6) | 7 (58.3) |  | 3 (27.3) | 6 (31.6) |  | 6 (50.0) | 3 (42.9) |  |
| Genes involved in alcohol metabolism |  |  |  |  |  |  |  |  |  |  |  |  |
| *ADH1B* (number of participants, %) |  |  | 0.35^b^ |  |  | 0.46^b^ |  |  | 0.82^b^ |  |  | 0.34^b^ |
| *1/*1 | 3 (13.0) | 0 (0.0) |  | 3 (14.3) | 1 (8.3) |  | 1 (9.1) | 1 (5.2) |  | 1 (8.3) | 0 (0.0) |  |
| *1/*2 | 7 (30.4) | 4 (25.0) |  | 5 (23.8) | 1 (8.3) |  | 1 (9.1) | 4 (21.1) |  | 3 (25.0) | 0 (0.0) |  |
| *2/*2 | 13 (56.5) | 12 (75.0) |  | 13 (61.9) | 10 (83.4) |  | 9 (81.8) | 14 (73.7) |  | 8 (66.7) | 7 (100) |  |
| *ALDH2* (number of participants, %) |  |  | 0.09^b^ |  |  | 1.00^b^ |  |  | 1.00^b^ |  |  | 1.00^b^ |
| *1/*1 | 14 (60.9) | 14 (87.5) |  | 18 (85.7) | 11 (91.7) |  | 10 (90.9) | 16 (84.2) |  | 9 (75.0) | 6 (85.7) |  |
| *1/*2 | 9 (39.1) | 2 (12.5) |  | 3 (14.3) | 1 (8.3) |  | 1 (9.1) | 3 (15.8) |  | 3 (25.0) | 1 (14.3) |  |
| *2/*2 | 0 (0.0) | 0 (0.0) |  | 0 (0.0) | 0 (0.0) |  | 0 (0.0) | 0 (0.0) |  | 0 (0.0) | 0 (0.0) |  |
| AQoLS (points, median, IQR) | 1.0 (2.5) | 2.0 (3.3) | 0.28^c^ | 1.0 (3.0) | 3.0 (2.8) | 0.60^c^ | 2.0 (4.0) | 5.0 (5.0) | 0.34^c^ | 6.0 (2.8) | 6.0 (4.0) | 0.99^c^ |
| Subjective view of health (number of participants, %) |  |  | 0.18^b^ |  |  | 0.64^b^ |  |  | 0.58^b^ |  |  | 0.12^b^ |
| Very healthy | 8 (34.8) | 7 (43.8) |  | 4 (19.0) | 4 (33.3) |  | 2 (18.2) | 3 (15.8) |  | 1 (8.3) | 3 (42.9) |  |
| Fairly healthy | 15 (65.2) | 7 (43.8) |  | 16 (76.2) | 8 (66.7) |  | 8 (72.7) | 16 (84.2) |  | 11 (91.7) | 4 (57.1) |  |
| Not so healthy | 0 (0.0) | 2 (12.5) |  | 1 (4.8) | 0 (0.0) |  | 1 (9.1) | 0 (0.0) |  | 0 (0.0) | 0 (0.0) |  |
| Not healthy | 0 (0.0) | 0 (0.0) |  | 0 (0.0) | 0 (0.0) |  | 0 (0.0) | 0 (0.0) |  | 0 (0.0) | 0 (0.0) |  |

N.D., not detect. ^a^Chi-square test. ^b^Fisher's exact probability test, ^c^Mann–Whitney U test.

Table S2 Numeric data on longitudinal changes from baseline values in non-alcoholic beverage and alcohol consumption, drinking frequency, and alcohol consumption on drinking days.

|  |  |  |  |  |  |  |  |  |  |  |  |  |  |  |  |  |  |  | Two-way ANOVA (*P*-value & effect size) | | |
| --- | --- | --- | --- | --- | --- | --- | --- | --- | --- | --- | --- | --- | --- | --- | --- | --- | --- | --- | --- | --- | --- |
|  |  |  | Week 4 | | | Week 8 | | | Week 12 | | | Week 16 | | | Week 20 | | |  | Interaction | Group | Time |
| ΔNon-alcoholic beverage consumption (mL/4 weeks) | | |  |  |  |  |  |  |  |  |  |  |  |  |  |  |  |  |  |  |  |
|  | Group 1 | Control | 84.8 | ± | 657.7 | 58.7 | ± | 402.5 | 52.2 | ± | 501.2 | -78.3 | ± | 339.4 | 57.0 | ± | 660.8 |  | p < 0.001  (η_p_^2^ = 0.394) | p < 0.001  (η_p_^2^ = 0.791) | p = 0.001  (η_p_^2^ = 0.403) |
|  |  | Intervention | 12457.8 | ± | 5192.6 | 12071.9 | ± | 5744.3 | 9165.6 | ± | 4315.5 | 6575.0 | ± | 4232.8 | 4437.5 | ± | 3647.5 |  |  |  |  |
|  | Group 2 | Control | -133.3 | ± | 649.9 | -233.3 | ± | 2195.1 | -469.1 | ± | 2245.4 | -450.0 | ± | 1750.5 | -550.0 | ± | 2130.6 |  | p < 0.001  (η_p_^2^ = 0.339) | p < 0.001  (η_p_^2^ = 0.485) | p < 0.001  (η_p_^2^ = 0.376) |
|  |  | Intervention | 9054.2 | ± | 5843.3 | 8866.7 | ± | 7719.3 | 8333.3 | ± | 8192.6 | 5454.2 | ± | 5551.0 | 1268.8 | ± | 3095.8 |  |  |  |  |
|  | Group 3 | Control | 256.4 | ± | 1106.7 | 1492.7 | ± | 4685.0 | 720.0 | ± | 2789.9 | 465.5 | ± | 1494.9 | 370.0 | ± | 1761.6 |  | p < 0.001  (η_p_^2^ = 0.150) | p < 0.001  (η_p_^2^ = 0.387) | p < 0.001  (η_p_^2^ = 0.178) |
|  |  | Intervention | 11756.6 | ± | 9254.0 | 10518.9 | ± | 7935.7 | 8786.8 | ± | 6143.7 | 5571.1 | ± | 5282.9 | 3676.3 | ± | 5013.2 |  |  |  |  |
|  | Group 4 | Control | -79.2 | ± | 770.6 | -354.2 | ± | 602.4 | -429.2 | ± | 743.3 | -312.5 | ± | 562.1 | -79.2 | ± | 928.0 |  | p < 0.001  (η_p_^2^ = 0.288) | p < 0.001  (η_p_^2^ = 0.742) | p = 0.003  (η_p_^2^ = 0.287) |
|  |  | Intervention | 13100.0 | ± | 3493.3 | 11000.0 | ± | 8900.1 | 8800.0 | ± | 6536.8 | 5400.0 | ± | 5503.9 | 4300.0 | ± | 5439.9 |  |  |  |  |
| ΔAlcohol consumption (%/4 weeks) | | |  |  |  |  |  |  |  |  |  |  |  |  |  |  |  |  |  |  |  |
|  | Group 1 | Control | -9.6 | ± | 37.4 | -13.1 | ± | 29.8 | -16.7 | ± | 23.7 | -22.2 | ± | 32.0 | -21.4 | ± | 29.3 |  | p = 0.092 (η_p_^2^ = 0.052) | p = 0.001  (η_p_^2^ = 0.256) | p = 0.969 (η_p_^2^ = 0.004) |
|  |  | Intervention | -46.9 | ± | 26.5 | -47.2 | ± | 23.2 | -46.8 | ± | 23.2 | -37.8 | ± | 30.2 | -39.6 | ± | 35.5 |  |  |  |  |
|  | Group 2 | Control | 0.1 | ± | 20.9 | 0.1 | ± | 27.8 | -1.2 | ± | 32.1 | -6.6 | ± | 28.4 | -6.0 | ± | 37.0 |  | p = 0.542 (η_p_^2^ = 0.025) | p < 0.001 (η_p_^2^ = 0.308) | p = 0.917 (η_p_^2^ = 0.008) |
|  |  | Intervention | -31.5 | ± | 22.5 | -39.3 | ± | 26.7 | -35.1 | ± | 28.6 | -30.4 | ± | 2.8 | -33.6 | ± | 26.5 |  |  |  |  |
|  | Group 3 | Control | -4.6 | ± | 25.4 | -12.4 | ± | 22.3 | -8.0 | ± | 20.4 | -2.0 | ± | 22.4 | -7.3 | ± | 35.0 |  | p = 0.780 (η_p_^2^ = 0.015) | p = 0.016 (η_p_^2^ = 0.192) | p = 0.614 (η_p_^2^ = 0.023) |
|  |  | Intervention | -25.5 | ± | 14.8 | -26.1 | ± | 23.7 | -21.3 | ± | 21.0 | -23.1 | ± | 16.7 | -21.8 | ± | 22.2 |  |  |  |  |
|  | Group 4 | Control | -12.4 | ± | 22.5 | -21.1 | ± | 24.6 | -9.5 | ± | 26.0 | -19.5 | ± | 29.2 | -23.4 | ± | 28.7 |  | p = 0.022 (η_p_^2^ = 0.154) | p = 0.965 (η_p_^2^ < 0.001) | p = 0.707 (η_p_^2^ = 0.031) |
|  |  | Intervention | -19.4 | ± | 15.7 | -5.5 | ± | 26.0 | -30.4 | ± | 22.3 | -15.2 | ± | 25.4 | -17.3 | ± | 13.4 |  |  |  |  |
| ΔDrinking frequency (days/4 weeks) | | |  |  |  |  |  |  |  |  |  |  |  |  |  |  |  |  |  |  |  |
|  | Group 1 | Control | -1.43 | ± | 3.10 | -1.78 | ± | 4.13 | -2.39 | ± | 4.10 | -3.43 | ± | 5.61 | -3.48 | ± | 5.01 |  | p = 0.383  (η_p_^2^ = 0.028) | p < 0.001  (η_p_^2^ = 0.347) | p = 0.862  (η_p_^2^ = 0.009) |
|  |  | Intervention | -8.94 | ± | 7.04 | -8.81 | ± | 6.13 | -8.31 | ± | 5.87 | -7.50 | ± | 6.65 | -8.81 | ± | 8.09 |  |  |  |  |
|  | Group 2 | Control | -0.40 | ± | 2.60 | -0.04 | ± | 3.07 | -0.14 | ± | 3.15 | -0.57 | ± | 3.46 | -1.62 | ± | 4.92 |  | p = 0.103 (η_p_^2^ = 0.060) | p = 0.012 (η_p_^2^ = 0.188) | p = 0.243 (η_p_^2^ = 0.043) |
|  |  | Intervention | -3.25 | ± | 5.39 | -5.67 | ± | 5.53 | -4.17 | ± | 5.42 | -3.67 | ± | 5.66 | -4.33 | ± | 5.48 |  |  |  |  |
|  | Group 3 | Control | -1.36 | ± | 3.23 | -2.64 | ± | 2.94 | -1.45 | ± | 2.21 | -1.36 | ± | 1.43 | -2.64 | ± | 4.90 |  | p = 0.539 (η_p_^2^ = 0.027) | p = 0.642 (η_p_^2^ = 0.008) | p = 0.395 (η_p_^2^ = 0.036) |
|  |  | Intervention | -3.16 | ± | 4.49 | -2.68 | ± | 4.44 | -1.58 | ± | 3.52 | -2.11 | ± | 2.47 | -2.26 | ± | 2.60 |  |  |  |  |
|  | Group 4 | Control | -0.17 | ± | 3.38 | -1.00 | ± | 2.73 | -0.67 | ± | 2.90 | -1.25 | ± | 3.84 | -2.50 | ± | 5.42 |  | p = 0.096 (η_p_^2^ = 0.108) | p = 0.131 (η_p_^2^ = 0.129) | p = 0.922 (η_p_^2^ = 0.013) |
|  |  | Intervention | -3.86 | ± | 4.95 | -3.00 | ± | 3.83 | -4.86 | ± | 4.78 | -3.43 | ± | 4.24 | -1.86 | ± | 2.97 |  |  |  |  |
| ΔAlcohol consumption on drinking days (%) | | |  |  |  |  |  |  |  |  |  |  |  |  |  |  |  |  |  |  |  |
|  | Group 1 | Control | -2.4 | ± | 29.3 | -1.8 | ± | 23.6 | -4.1 | ± | 15.7 | -0.5 | ± | 32.8 | -4.8 | ± | 19.2 |  | p = 0.695  (η_p_^2^ = 0.015) | p = 0.905  (η_p_^2^ < 0.001) | p = 0.805  (η_p_^2^ = 0.011) |
|  |  | Intervention | -10.9 | ± | 23.8 | -3.6 | ± | 35.4 | -4.8 | ± | 47.3 | -1.5 | ± | 42.5 | 2.3 | ± | 49.7 |  |  |  |  |
|  | Group 2 | Control | 1.3 | ± | 16.0 | 0.8 | ± | 22.0 | -2.8 | ± | 21.4 | -5.3 | ± | 23.7 | -1.8 | ± | 33.0 |  | p = 0.851 (η_p_^2^ = 0.011) | p = 0.122 (η_p_^2^ = 0.075) | p = 0.980 (η_p_^2^ = 0.004) |
|  |  | Intervention | -14.1 | ± | 28.2 | -17.3 | ± | 37.9 | -14.6 | ± | 30.4 | -13.3 | ± | 40.2 | -13.9 | ± | 32.3 |  |  |  |  |
|  | Group 3 | Control | 1.5 | ± | 19.7 | 0.0 | ± | 20.3 | -0.5 | ± | 22.0 | 4.4 | ± | 21.8 | 11.7 | ± | 18.6 |  | p = 0.548 (η_p_^2^ = 0.027) | p = 0.004 (η_p_^2^ = 0.259) | p = 0.136 (η_p_^2^ = 0.060) |
|  |  | Intervention | -11.7 | ± | 14.4 | -18.0 | ± | 17.6 | -15.6 | ± | 19.6 | -14.4 | ± | 16.0 | -12.8 | ± | 22.8 |  |  |  |  |
|  | Group 4 | Control | -10.9 | ± | 22.0 | -16.7 | ± | 23.5 | -6.2 | ± | 27.6 | -14.6 | ± | 22.1 | -14.5 | ± | 22.9 |  | p = 0.025 (η_p_^2^ = 0.150) | p = 0.323 (η_p_^2^ = 0.058) | p = 0.517 (η_p_^2^ = 0.046) |
|  |  | Intervention | -0.6 | ± | 20.6 | 7.4 | ± | 28.3 | -11.5 | ± | 15.5 | -1.1 | ± | 27.0 | -9.4 | ± | 9.2 |  |  |  |  |
